# Dexmedetomidine combined with etomidate or emulsified isoflurane for induction reduced cardiopulmonary response in dogs

**DOI:** 10.1371/journal.pone.0208625

**Published:** 2018-12-07

**Authors:** Chao Liu, Tingting Lin, Zhenlei Zhou

**Affiliations:** College of Veterinary Medicine, Nanjing Agricultural University, Nanjing, Jiangsu, China; University of Bari, ITALY

## Abstract

To investigate the effects of etomidate, emulsified isoflurane, and their combination with dexmedetomidine on physiological parameters, electrocardiogram (ECG) results, and the quality of induction and recovery during isoflurane maintenance anaesthesia. 5 mixed-breed dogs received each of four treatments: etomidate (E group); emulsified isoflurane (EI group); both dexmedetomidine and etomidate (DE group); or both dexmedetomidine and emulsified isoflurane (DEI group). All drugs were IV injection administered for induction, followed by 1.5 MAC (minimal alveolar concentration) of isoflurane to maintain anaesthesia. Rectal temperature (RT), respiratory rate (RR), heart rate (HR), mean arterial pressure (MAP), and ECG were measured at baseline, 0, 5, 10, 20, 40, and 60 minutes after intubation. The quality of induction and recovery was evaluated for all dogs. All the anaesthetic procedures provided good conditions for induction of anaesthesia. The quality of induction and recovery in the E group was worse than other groups. The decrease of RR in the E and DE groups was stronger than that in the EI and DEI groups. The dogs in the E group had the most significant prolongation of the Q-T interval and changes in the S-T segment. Deviation and extension of the S-T segment were noted in the El group. The dogs in the DE and DEI groups had fewer changes in the ECG results than those in the E and EI groups. The addition of dexmedetomidine caused less effect on cardiopulmonary parameters and the ECG results than either etomidate or emulsified isoflurane alone. Thus, etomidate or emulsified isoflurane in combination with dexmedetomidine may be useful clinically for the induction of anaesthesia.

## Introduction

Isoflurane is commonly used as an inhalation anaesthetic in the veterinary clinic; it is considered safe for the cardiovascular system and has little influence on hepatic metabolism [1]. The most prominent adverse effect during isoflurane anaesthesia is respiratory depression, which can result in hypercapnia and acidosis because of decreased respiration rate [2]. Opioids, α-2 agonists, dissociative anaesthetics, and diazepam were used in dogs as a single drug treatment or as part of pre-anaesthetic drug combinations to provide analgesia and to reduce the adverse effects and the amount of isoflurane anaesthetic required [3, 4].

Dexmedetomidine, an α-2 adrenergic receptor agonist, can provide sedative, analgesic, and anti-anxiety effects [5]. Dexmedetomidine suppresses the nociceptive reflex responses after electrical stimulation and might reduce the risk of developing post-operative chronic pain [6]. It has been reported that dexmedetomidine has bronchoprotective effects and reduces oxygen consumption, carbon dioxide production, and energy consumption [7, 8]. The disadvantages of dexmedetomidine treatment include bradycardia, hypotension, nausea, and dryness of mouth [9].

The clinical use of etomidate for anaesthesia induction was investigated in 1973 [10]. Etomidate demonstrates minimal cardiopulmonary and respiration adverse reactions, and it is suitable for hemodynamically unstable patients [11]. When anaesthesia is induced with etomidate, 50–80% of patients who are not premedicated experience myoclonus [12]. Pre-treatment with some agents such as sufentanil can reduce the incidence of myoclonus during etomidate-induced anaesthesia without any harmful side effects [13].

In 1995, a study using an intravenous (IV) administration of emulsified isoflurane (EI) in mice demonstrated that it can induce and maintain effective anaesthesia [14]. IV injection of EI requires significantly less isoflurane (80% less for induction, 20% less for maintenance) to obtain the same anaesthetic effect, and it does not affect the awakening time and blood partial pressure; these qualities have promoted the use of EI for organ protection in clinical fields [15, 16].

The use of EI for anaesthesia induction in the veterinary clinic still requires much clinical trial support. It has been reported that if anaesthetic premedications such as dexmedetomidine were administered in conjunction with the induction anaesthetic, then the quality and speed of the induction would greatly improve [17]. Thus, the purpose of the present study was to evaluate the effects of etomidate, EI, or their combination with dexmedetomidine on the cardiopulmonary system, ECG results, and the quality of induction and recovery during isoflurane maintenance anaesthesia in dogs.

## Materials and methods

### Animals

Five mixed-breed dogs were used in this study. The dogs weighed 9.2 ± 0.6 kg and were aged 12 to 36 months. All dogs were confirmed to be healthy by clinical examination, including blood routine examination, blood biochemistry, and ECG tests; they did not exhibit symptoms of any disease. Experimental animal welfare and ethics committee of Nanjing Agricultural University approved this research. The approval number is PTD2018027.

### Preparation of emulsified isoflurane (EI)

EI (8%) was prepared as described previously by adding 18.4 mL 30% Intralipid (Sino-Swed Pharmaceutical Corp. Ltd) and 1.6 mL liquid isoflurane (Hebei Yipin Pharmaceutical Co., Ltd.) to a 20-mL glass ampoule [18]. The ampoule was sealed using an alcohol blowtorch. The ampoules were placed on a vortex mixer (Ika vortex genius 3) and vigorously shaken for 15 min to completely mix isoflurane and Intralipid. Before treatment, the stability of the 8% EI was checked. Previous studies have found that 8% EI remained stable for 6 months at room temperature [19].

### Study protocol

5 dogs fasted for at least 8 hours before the experiment and were provided with water at all times until the time of drug administration. The dogs were placed in the operating room for 30~60 minutes before pre-medication. The body weight and baseline parameters of each dog were measured. 5 Dogs received each of four treatments, with at least 2 weeks washout time before the next treatment The treatment groups were: E group, etomidate (3 mg/kg, Jiangsu Nhua Pharm Group Co., Ltd.); EI group, emulsified isoflurane (1 mL/kg); DE group, dexmedetomidine (3 μg/kg, Pfizer Pharmaceutical Co., Ltd, NY, USA); and etomidate (2 mg/kg); DEI group, dexmedetomidine (3 μg/kg) and emulsified isoflurane (0.5 mL/kg) [20–23]. In DE and DEI groups, dexmedetomidine was administered IV at least 3 minutes prior to induction of anaesthesia.

A bolus dose of atropine (0.04 mg/kg; Wuhu Kangqi Pharmaceutical Co., Ltd.) was intramuscular (IM) administered at 15 minutes prior to induction in all dogs. Induced anaesthetic was IV administered in 10 seconds. In the DE and DEI groups, dexmedetomidine was administered IV at least 3 minutes prior to induction of anaesthesia. All dogs were positioned in the right lateral position. After tracheal intubation, a standard circle anaesthetic system (Stinger Streamline Small Animal, Datex Ohmeda Isotec 5, AAS) was used. During the experiment, anesthetized animals were breathing spontaneously using the circle system (VIC), which used low-flow inhalation anesthesia. The dogs were administered isoflurane (1.5 MAC) and oxygen (30 mL/kg /min) to maintain 60 minutes of anaesthesia.

Noninvasive arterial blood pressure (6.5 cm width cuff placed on the thoracic limb above the carpus), rectal temperature (RT), haemoglobin oxygen saturation (SpO2) and heart rate (HR) were monitored using a multi-parameter physiological monitor (Datex-Ohmeda S/5; Datex-Ohmeda Division Instrumentarium Corp., Finland). The systolic, diastolic, and mean arterial blood pressures (SAP, DAP, and MAP, respectively) were measured at every time point. The respiratory rate (RR) was recorded by counting chest movements. All dogs were subjected to standard 6-lead electrocardiograms, and The ECG device (ECG- 3306G) were recorded at a paper speed of 25 mm/sec with an amplitude of 1 mV/cm. Hair was clipped from the areas of electrode attachment and the skin was covered with gel and then crocodile electrodes were situated almost above knee joint in the hind legs and elbow joint in the front legs. ECGs were recorded with I, II, III, aVR, aVL and AVF derivations. Measurements of the amplitude (mV) of P, R and T waves, and the durations (ms) of P and T waves, P-R, Q-T, and QRS intervals were performed in lead II. 3 cardiac complexes were the measurements averaged at each time point. The data were obtained at baseline and 0 (Immediately after intubation), 5, 10, 20, 40, and 60 minutes after intubation.

A single observer unaware of group allocation assessed and noted the scale. The quality of induction and recovery was scored using a standardized scale (S1 and S2 Tables) [24, 25]. Details of any abnormal behaviour or adverse reactions such as ataxia, vocalizations, and convulsions among others were recorded. An assistant recorded the time to extubation, palpebral reflex recovery, raising the head, and standing up.

### Statistical analysis

All variables were compared to the baseline and among the groups. All data were presented as mean ± standard deviation (SD). Statistical analyses were performed with SPSS software. The differences among groups were determined with one-way analysis of variance (ANOVA, Dunnet’s T3). Statistical differences were considered significant at *P < 0*.*05*.

## Results

Good induction results were observed in all the 4 groups. There were no significant differences in sedation, intubation scores, and time to palpebral reflex recovery (Table 1).

**Table 1:** Comparison of induction and recovery scores, and the induction and recovery time in the 4 groups (E, EI, DE, and DEI) after intravenous administration and maintenance anaesthesia with isoflurane (1.5 MAC) for 60 min.

	E	EI	DE	DEI
Induction time of anaesthesia (min)	0.76±0.22^b^	1.51±0.86	0.65±0.11^b^	0.78±0.08^b^
Sedation score	3.00±0.00	3.00±0.00	3.00±0.00	3.00±0.00
Intubation score	0.75±0.50	0.60±0.89	0.25±0.50	0.50±0.58
Induction quality score	1.00±0.00	0.60±0.54	0.20±0.44^a^	0.20±0.44^a^
Recovery quality score	2.20±0.84^C^	1.50±0.84	1.40±0.89	1.00±0.00
Time to palpebral reflex recovery (min)	1.79±1.16	1.92±0.74	1.61±1.25	1.41±0.51
Time to extubation (min)	2.14±1.43	4.59±2.43	5.77±1.88^a^	3.87±2.10
Time to raising the head (min)	4.31±1.80	4.50±1.80	6.64±2.05	4.35±2.14
Time to first stand-up (min)	7.48±2.76	5.29±1.64	7.39±1.97	5.25±2.10

The values are presented as mean ± standard deviation.

^a^ Significantly different from E group (*P*<0.05).

^b^ Significantly different from EI group (*P*<0.05).

^c^ Significantly different from DEI group (*P*<0.05).

The quality of induction and recovery in the E group was worse than that in the other groups. Individual dogs in the E group exhibited muscle spasms and laryngeal reflex during induction and showed excitement or paddling during recovery. The time of induction anaesthesia in the EI group was significantly longer than that in the other groups (*P*<0.05). The time of extubation and the time to raise the head was significantly longer in the DE group than in the E, EI, and DEI groups (*P*<0.05). The dogs in EI and DEI groups had a shorter time to stand up than those in the E and DE groups (*P*<0.05).

The SpO_2_ values did not show a significant change in any group during the 60 minutes of isoflurane maintenance anaesthesia. The RT in the 4 groups decreased with the prolongation of anaesthesia time. The HR in the E and EI groups increased by 0 to 10 minutes more than that in the DE and DEI groups, and this difference was significant (*P*<0.05). The 4 induction treatments significantly reduced the RR, and the DE group showed the strongest reduction (*P*<0.05). The SAP, DAP, and MAP in all groups decreased during isoflurane maintenance anaesthesia. However, SAP, DAP, and MAP in the DE and DEI groups increased during 0 to 5 minutes of intubation. There were significant differences in SAP, DAP, MAP, RT, and RR in all groups in 40 to 60 minutes of intubation when compared with that at the baseline. (*P*<0.05) (Table 2).

**Table 2:** Comparison of the cardiopulmonary effects in the 4 groups (E, EI, DE, and DEI) by intravenous administration and maintenance anaesthesia with isoflurane (1.5 MAC) for 60 min.

	Group	Baseline	0	5	10	20	40	60
SAP (mmHg)	E	127±11	121±7	103±10*	99±9*	93±8*	95±13*	99±18*
	EI	119±5	102±9*	97±11*	95±11*	92±6*	89±6*	91±12*
	DE	130±9	141±17	130±20	125±21	113±11	96±11*	89±5*
	DEI	131±9	140±20	132±22	123±30	106±15*	90±8*	94±9*
DAP (mmHg)	E	59±24	67±11	46±5	45±10	41±5*	44±8*	46±14.
	EI	57±15	48±6	48±15	48±12	44±5	43±9	48±9
	DE	80±6	97.0±29	89±25	92±33	79±23	51±8*	49±11*
	DEI	72±11	103±21*	94±23*	83±31	60±15.	47±7*	49±6*
MAP (mmHg)	E	95±7.5	85±9	65±6	63±9*	59±5*	61±9*	62±13*
	EI	83±10	66±5*	66±16*	69±12	60±5*	58±8*	63±10*
	DE	107±5	114±20	107±24	106±12	86±17	64±8*	60±9*
	DEI	102±11	119±21*	110±23	102±31	75±15*	61±5*	63±7*
RT(°C)	E	38.3±0.3	38.2±0.3	37.9±0.1	37.8±0.1	37.7±0.4	37.6±0.6*	37.4±0.6*
	EI	39.0±0.5	38.6±0.4	38.5±0.3	38.4±0.3*	38.2±0.3*	37.8±0.4*	37.7±0.6*
	DE	38.7±0.2	38.6±0.2	38.7±0.3	38.6±0.3	38.5±0.4	38.2±0.4*	37.8±0.2*
	DEI	39.0±0.2	38.7±0.6	38.7±0.6	38.6±0.5	38.5±0.5*	38.5±0.5*	38.2±0.6*
HR beats·min^-1^	E	97±18	123±4*	123±9*	123±14*	110.4±9	97±12	102±10
	EI	102±15	124±18*	126±7*	125±9*	111±18	109±16	115±14
	DE	101±14	103±18	105±23	118±25	108±18	103±8	97±5
	DEI	115±24	118±22	114±17	109±14	106±18	102±19	96±20
RR Respiration·min^-1^	E	20±4	9±3*	11±5*	12±7*	8±2*	10±3*	14±7
	EI	30±2	10±2*	8±2*	10±3*	13±3*	15±6*	16±7*
	DE	21±5	6±2*	9±4*	9±3*	9±3*	12±5*	11±2*
	DEI	24±4	12±2*	13±3*	13±2*	11±1*	13±1*	14±2*
SpO_2_	E	—	97.8±0.8	97.2±1.5	97.4±1.1	97.8±0.83	98.8±1.0	99.2±0.8
	EI	—	97.4±0.54	98.0±0.7	98.4±1.1	98.0±1.0	98.0±1.0	98.0±1.2
	DE	—	98.6±2.1	99.0±1.0	98.6±1.1	98.6±0.5	99.0±0.2	99.0±1.2
	DEI	—	98.6±1.3	98.0±0.9	97.6±1.8	98.6±0.8	98.6±0.8	98.7±0.9

The values are presented as mean ± standard deviation. SAP, systolic arterial pressure; DAP, diastolic arterial pressure; MAP, mean arterial pressure; RT, rectal temperature; HR, heart rate; RR, respiratory rate; SpO2, haemoglobin oxygen saturation. Time points: baseline, 0, 5, 10, 20, 40, and 60 min after maintenance anaesthesia. "—", not available;

*Significantly different from the baseline within the group (*P* < 0.05).

Table 3 shows the changes in the ECG results. The P-wave duration slightly increased in the EI group, while the P-wave amplitude increased in the E and DE groups and did not change in the DEI group between 0 and 10 minutes during isoflurane maintenance anaesthesia (*P* > 0.05). The duration of the QRS complex in the E group significantly decreased compared with the baseline (*P* < 0.05).

**Table 3:** The ECG changes in the 4 groups with intravenous administration of E, EI, DE, and DEI and maintenance anaesthesia with isoflurane (1.5 MAC) for 60 min.

	Group	Base	0	5	10	20	40	60
P-wave duration (s)	E	0.040±0.000	0.038±0.002	0.037±0.002	0.037±0.002	0.039±0.002	0.038±0.002	0.038±0.002
	El	0.036±0.005	0.043±0.007	0.042±0.005	0.038±0.004	0.043±0.007	0.039±0.003	0.037±0.004
	DE	0.040±0.000	0.039±0.004	0.038±0.005	0.036±0.003	0.037±0.003	0.038±0.005	0.037±0.002
	DEl	0.038±0.004	0.038±0.005	0.035±0.005	0.039±0.005	0.040±0.003	0.036±0.003	0.038±0.002
P-wave amplitude (mV)	E	0.19±0.07	0.23±0.08	0.22±0.08	0.22±0.09	0.18±0.06	0.16±0.05	0.17±0.05
	El	0.15±0.06	0.18±0.07	0.17±0.05	0.18±0.04	0.17±0.04	0.17±0.03	0.17±0.04
	DE	0.17±0.03	0.21±0.05	0.21±0.04	0.20±0.04	0.18±0.05	0.17±0.05	0.17±0.02
	DEl	0.14±0.04	0.15±0.04	0.16±0.04	0.16±0.03	0.16±0.03	0.16±0.04	0.15±0.04
QSR-wave duration (s)	E	0.054±0.002	0.043±0.006*	0.045±0.006*	0.046±0.007*	0.044±0.003*	0.045±0.004*	0.044±0.006*
	El	0.050±0.000	0.048±0.008	0.048±0.008	0.047±0.005	0.048±0.007	0.046±0.006	0.046±0.007*
	DE	0.055±0.004	0.046±0.006	0.045±0.005*	0.047±0.008	0.049±0.008	0.047±0.006	0.048±0.007
	DEl	0.058±0.004	0.048±0.009	0.048±0.011	0.047±0.012	0.048±0.010	0.046±0.008*	0.049±0.007
R-wave amplitude (mV)	E	1.15±0.70	1.16±0.48	1.06±0.48	1.15±0.41	1.15±0.46	1.23±0.52	1.28±0.61
	El	1.10±0.39	1.29±0.46	1.35±0.46	1.26±0.47	1.26±0.59	1.30±0.66	1.34±0.65
	DE	0.96±0.55	1.14±0.47	1.08±0.45	1.06±0.50	0.93±0.55	0.78±0.59	0.70±0.54
	DEl	1.28±0.56	1.52±0.54	1.48±0.50	1.39±0.45	1.22±0.45	1.08±0.55	1.00±0.51
T-wave duration (s)	E	0.074±0.008	0.093±0.018	0.095±0.014	0.104±0.018*	0.114±0.012*	0.118±0.008*	0.121±0.013*
	El	0.063±0.027	0.075±0.030	0.079±0.017	0.074±0.021	0.085±0.023	0.088±0.018	0.092±0.018
	DE	0.066±0.009	0.090±0.013	0.084±0.018	0.094±0.016*	0.095±0.024*	0.091±0.022*	0.094±0.018*
	DEl	0.071±0.012	0.089±0.008	0.094±0.019	0.081±0.013	0.077±0.020	0.083±0.016	0.082±0.017
T-wave amplitude(mV)	E	0.27±0.06	0.50±0.19	0.51±0.18	0.59±0.23*	0.75±0.22*	0.76±0.21*	0.75±0.19*
	EI	0.23±0.03	0.38±0.09	0.33±0.08	0.33±0.11	0.37±0.11	0.41±0.12*	0.39±0.14*
	DE	0.22±0.05	0.43±0.13*	0.39±0.13	0.41±0.18*	0.42±0.16*	0.40±0.15	0.40±0.13*
	DEI	0.35±0.20	0.50±0.21	0.44±0.19	0.42±0.17	0.43±0.15	0.42±0.15	0.41±0.15
P-R interval (s)	E	0.099±0.005	0.086±0.018	0.079±0.011*	0.080±0.010*	0.082±0.010*	0.085±0.012	0.092±0.011
	EI	0.090±0.007	0.084±0.008	0.086±0.010	0.082±0.007	0.089±0.009	0.086±0.009	0.083±0.010
	DE	0.098±0.009	0.101±0.016	0.092±0.014	0.099±0.015	0.098±0.012	0.097±0.012	0.094±0.006
	DEI	0.092±0.008	0.090±0.011	0.089±0.008	0.090±0.010	0.092±0.010	0.091±0.010	0.094±0.010
Q-T interval (s)	E	0.216±0.019	0.223±0.011	0.225±0.010	0.228±0.009	0.241±0.007*	0.253±0.014*	0.255±0.017*
	EI	0.200±0.000	0.231±0.038	0.229±0.041	0.223±0.047	0.233±0.041	0.219±0.024	0.225±0.020
	DE	0.212±0.018	0.219±0.015	0.214±0.008	0.213±0.013	0.222±0.008	0.233±0.010*	0.237±0.011*
	DEI	0.205±0.011	0.210±0.020	0.214±0.019	0.218±0.017	0.222±0.012	0.228±0.019*	0.227±0.016*
S-T segment (s)	E	0.090±0.012	0.087±0.009	0.085±0.010	0.079±0.011	0.083±0.012	0.083±0.006	0.091±0.010
	EI	0.075±0.025	0.087±0.017	0.085±0.017	0.090±0.008	0.079±0.011	0.087±0.009	0.089±0.014
	DE	0.082±0.015	0.074±0.016	0.080±0.012	0.071±0.014	0.078±0.029	0.085±0.018	0.094±0.024
	DEI	0.080±0.024	0.074±0.027	0.073±0.027	0.090±0.020	0.098±0.024	0.095±0.027	0.094±0.023

The values are presented as mean ± standard deviation. Time points: baseline, 0, 5, 10, 20, 40, and 60 minutes after maintenance anaesthesia.

* Significantly different from the baseline within the group (*P* < 0.05).

The duration of the QRS complex in other groups also decreased but less than in the E group. The R-wave amplitude did not change when compared with the baseline in all groups (*P* > 0.05). The duration and amplitude of the T-wave increased in all groups when compared with the baseline and increased significantly in the E and DE groups (*P* <0.05). The P-R interval in the E group was significantly shorter between 5 and 10 minutes. The 4 types of treatment caused a prolongation of the Q-T interval, which was most significant in the E group. An S-T segment depression or deviation was found in the E group. One dog in the EI group had an abnormal S-T segment change during anaesthesia.

**Fig 1:**
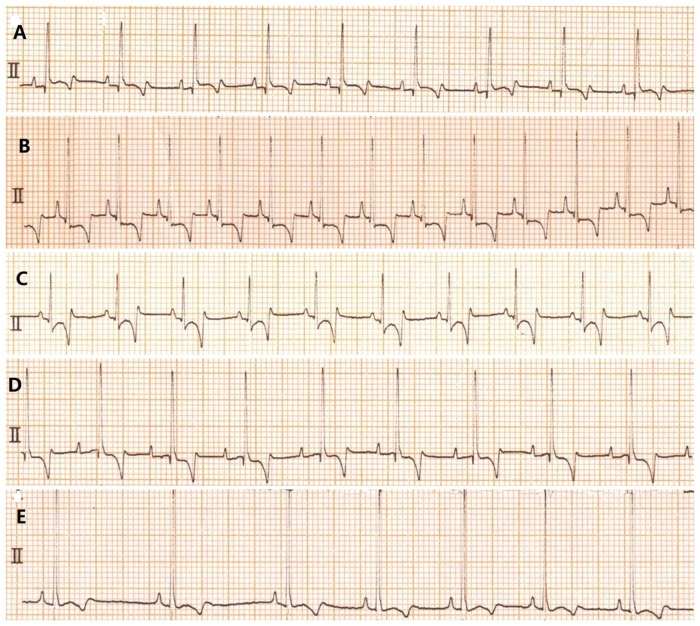
A: The normal ECG of dogs. B: After 5 minutes of isoflurane anaesthesia, the E group showed prolongation of the Q-T interval, S-T segment depression, and sinus tachycardia (150 beats∙ min^-1^). C: After 5 minutes of isoflurane anaesthesia, the EI group had S-T segment changes. D: After 5 minutes of isoflurane anaesthesia, the DE group had prolongation of the P-R interval and first-degree A-V block. E: After 5 minutes of isoflurane anaesthesia, the DEI group had sinus arrhythmia and the R-wave amplitude increased.

The E group had significant sinus tachycardia after the induction of anaesthesia. In the DE group, only 1 dog exhibited first degree atrioventricular block during 0 to 5 min. There was sinus arrhythmia in the DEI group during 0 to 5 minutes (Fig 1).

## Discussion

In this study, 4 types of induction methods provided good induction effects with fast onset of action and few adverse reactions. All the treatments ensured the smooth progress of endotracheal intubation. The results were similar to those obtained in other studies using etomidate and EI [16, 26].

Etomidate administration result in myoclonus, excitement or paddling during recovery, and post-operative agitation. It has been proposed that myoclonus after etomidate administration occurs due to subcortical disinhibition [12, 27]. Some drugs such as midazolam, fentanyl, and dexmedetomidine have been recommended to reduce the incidence of myoclonus after etomidate administration [28]. In the present study, we did not observe obvious adverse reactions in dogs of the DE and DEI groups.

The dogs in the EI and DEI groups had a shorter time to stand up than those in the E and DE groups. EI intravenous infusion is mainly eliminated through the lungs [16]. Etomidate is primarily metabolized by esterase in the liver, and about 75% of the injected etomidate is excreted in urine, 10% in bile, and 13% in faeces [29]. In our study treatments, EI allowed an earlier standing-up time than etomidate. DE and E groups had a long standing-up time, which was more likely to be superposed with the 1.5 MAC isoflurane maintenance and finally led to more severe respiratory suppression. And respiratory depression delayed the elimination of isoflurane and stand-up time. The results showed that etomidate and dexmedetomidine administration produced a slower metabolic rate than EI.

The blood pressure increased during 0 to 5 minutes in dogs of the DE and DEI groups. Some studies reported that isoflurane anaesthesia leads to hypotension [30], and EI reduces blood pressure [31]. Therefore, the increase in blood pressure in this study was a result of dexmedetomidine administration, which is accordance with a report that the addition of dexmedetomidine during isoflurane anaesthesia increased MAP, SAP, and DAP [32].

In this study, significant increase in HR was found in dogs of the E and EI groups, except DE and DEI groups. This is due to the administration of dexmedetomidine causing vasoconstriction in the pulmonary and systemic circulation and thereby decreasing HR [32], which reduced the effects of EI and etomidate. The 4 anaesthesia combination treatments demonstrated significant respiratory depression. This inhibitory effect was the most significant in the DE group, which might be because the combination of 2 drugs increased the inhibitory effect.

Electrocardiogram studies have been used to examine the effect of endotracheal intubation on ECG patterns during halothane anaesthesia since 1962 [33]. The ECG not only helps to diagnose myocardial infarction but also provides information on infarct location, reperfusion situation, and prognosis [34]. In the present study, T-wave amplitude and duration increased in the 4 groups. The T-wave morphology is variable in dogs, and the T-wave changes are very limited for the clinical diagnosis of dogs when compared with that in humans [35]. Etomidate administration led to a significant prolongation of the Q-T interval, and the S-T segment deviation or depression. Emulsified isoflurane administration caused extension of the S-T segment. The S-T segment changes occurred in all treatments during isoflurane anaesthesia. Sprung et al studied isoflurane anaesthesia and found that it induced S-T changes and ECG changes consistent with myocardial ischemia [36]. Tatekawa et al showed that maintaining the mean arterial pressure at 80 mmHg was sufficient to prevent myocardial ischemic changes in dogs [37]. In our study, the MAP in the E and EI groups frequently decreased below 80 mmHg. After a combination of dexmedetomidine with etomidate or EI induction, the negative impact on MAP and ECG reduced during isoflurane maintenance anaesthesia.

One dog in the DE group developed first-degree arterio-venous (A-V) block during anaesthesia, and the DEI group showed slight sinus arrhythmia on the ECG. Previous studies have reported that the administration of dexmedetomidine as a pre-anaesthetic agent causes cardiac arrest [5]. The initial use of antimuscarinic drugs can prevent bradycardia arrhythmia associated with α-2 agonists [38]. In the present study, a clinically low dose of dexmedetomidine (3 μg/kg) was only related to single A-V block or arrhythmia, and no bradycardia or cardiac arrest developed.

## Conclusions

In this study, all administered drugs demonstrated good anaesthetic effects. Dexmedetomidine decreased the use of etomidate or EI needed for induction. During isoflurane maintenance anaesthesia, the S-T segment changes suggested the occurrence of myocardial ischemia. Combined administration of dexmedetomidine with etomidate or EI had less influence on the circulation, respiratory system, and ECG than the use of the either agents alone. The combination of etomidate or EI with dexmedetomidine may represent a useful protocol for veterinary clinical application in dogs.

## Supporting information

S1 Table Quality scoring systems. (DOC)

S2 Table Description of recovery score categories. (DOC)
