# Supplementary material for: Dexmedetomidine combined with etomidate or emulsified isoflurane for induction reduced cardiopulmonary response in dogs
Source: PLoS One. 2018 Dec 7;13(12):e0208625. doi: 10.1371/journal.pone.0208625 (PMC6285997; doi:10.1371/journal.pone.0208625)
Supplement: S1 Table — (DOC) [file pone.0208625.s001.doc]

S1 Table: Quality scoring systems.

| Item | Score | Categories | Description |
| --- | --- | --- | --- |
| Sedation | 0 | No sedation | No visible signs of sedation |
|  | 1 | Mild | Quieter, but still bright and active |
|  | 2 | Moderate | Quiet, reluctant to move, ataxic, able to walk |
|  | 3 | Profound | Unable to walk unaided |
| Tracheal intubation | 0 | Smooth | No swallowing, coughing, tongue/jaw movement |
|  | 1 | Fair | Some tongue movement, slight cough |
|  | 2 | Poor | Marked tongue/jaw movement, swallowing |
|  | 3 | Very poor | As 2 but requiring additional induction agent |
| Quality of anaesthetic induction | 0 | Smooth | Without excitement |
|  | 1 | Fair | Slight excitement/muscle twitching/limb movement |
|  | 2 | Poor | Marked excitement/muscle twitching/limb movement |
|  | 3 | Very poor | As 2 plus vocalization |
