# Supplementary material for: Dexmedetomidine combined with etomidate or emulsified isoflurane for induction reduced cardiopulmonary response in dogs
Source: PLoS One. 2018 Dec 7;13(12):e0208625. doi: 10.1371/journal.pone.0208625 (PMC6285997; doi:10.1371/journal.pone.0208625)
Supplement: S2 Table — (DOC) [file pone.0208625.s002.doc]

S2 Table： Description of recovery score categories.

| Categories | Description |
| --- | --- |
| 1 | Very smooth, no excitement, vocalization, trembling or vomiting. No convulsions |
| 2 | Quite smooth, a little excitement. No paddling, vocalization, trembling or vomiting. No convulsions |
| 3 | Moderately smooth with excitement. Some paddling, vocalization, trembling or vomiting observed. No convulsions |
| 4 | Not smooth and with excitement. Paddling, vocalization, trembling or vomiting observed. No convulsions |
| 5 | Extreme excitement observed with aggression, vocalization, violent movements or convulsions observed. |
